# A mitochondrially targeted compound delays aging in yeast through a mechanism linking mitochondrial membrane lipid metabolism to mitochondrial redox biology^☆^

**DOI:** 10.1016/j.redox.2014.01.011

**Published:** 2014-01-23

**Authors:** Michelle T. Burstein, Vladimir I. Titorenko

**Affiliations:** Department of Biology, Concordia University*,* Montreal, Quebec, Canada

**Keywords:** CL, cardiolipin, IMM, inner mitochondrial membrane, LCA, lithocholic acid, MLCL, monolysocardiolipin, OMM, outer mitochondrial membrane, PA, phosphatidic acid, PC, phosphatidylcholine, PG, phosphatidylglycerol, PE, phosphatidylethanolamine, PS, phosphatidylserine, ROS, reactive oxygen species, Aging, Anti-aging natural compounds, Mitochondrial lipids, Mitochondrial redox biology, Mitochondrial respiration, Mitochondrial reactive oxygen species

## Abstract

A recent study revealed a mechanism of delaying aging in yeast by a natural compound which specifically impacts mitochondrial redox processes. In this mechanism, exogenously added lithocholic bile acid enters yeast cells, accumulates mainly in the inner mitochondrial membrane, and elicits an age-related remodeling of phospholipid synthesis and movement within both mitochondrial membranes. Such remodeling of mitochondrial phospholipid dynamics progresses with the chronological age of a yeast cell and ultimately causes significant changes in mitochondrial membrane lipidome. These changes in the composition of membrane phospholipids alter mitochondrial abundance and morphology, thereby triggering changes in the age-related chronology of such longevity-defining redox processes as mitochondrial respiration, the maintenance of mitochondrial membrane potential, the preservation of cellular homeostasis of mitochondrially produced reactive oxygen species, and the coupling of electron transport to ATP synthesis.

## Highlights

• Lithocholic bile acid (LCA) delays aging in yeast.
• LCA enters yeast cells and accumulates mainly in the inner mitochondrial membrane.
• LCA elicits major changes in the composition of mitochondrial membrane lipids.
• These changes in membrane lipidome alter mitochondrial redox processes.
• These alterations in mitochondrial redox biology delay cellular aging.

## Graphical abstract

**Figure:**
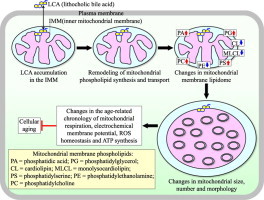

A body of evidence supports the notion that mitochondria regulate cellular aging in evolutionarily distant eukaryotic organisms [1–3]. These organelles compartmentalize various redox processes known to be essential for establishing the rate of cellular and organismal aging. Such longevity-defining redox processes in mitochondria include the coupling of electron transport to ATP synthesis, modulation of mitochondrial membrane potential, maintenance of cellular homeostasis of reactive oxygen species (ROS), and formation and release of certain metabolites and macromolecules that can set off a pro- or anti-aging cellular pattern [2,4–10]. Several small molecules have been shown to delay cellular and organismal aging in eukaryotes across phyla by modulating some of the mitochondria-confined redox processes. After being sorted to the mitochondrial matrix, the inner mitochondrial membrane (IMM) or the outer mitochondrial membrane (OMM), these anti-aging pharmaceuticals act as rechargeable antioxidants that attenuate oxidative damage to membrane proteins and/or phospholipids [11–17]. Our recent study revealed a previously unknown mechanism of delaying cellular aging by a mitochondrially targeted natural compound which specifically impacts mitochondrial redox biology [18]. The name of this compound is lithocholic acid (LCA), a bile acid which we identified in a high-throughput chemical genetic screen for small molecules extending longevity of chronologically aging yeast [19]. In the mechanism that we discovered, an exogenously added LCA enters yeast cells and accumulates mostly in the IMM; a minor portion of this bile acid is also confined to the OMM (Fig. 1). The pools of LCA accumulated in both mitochondrial membranes elicit an age-related remodeling of phospholipid synthesis and movement within the IMM and OMM (Fig. 1). Such specific remodeling of mitochondrial phospholipid dynamics progresses with the chronological age of a yeast cell and ultimately causes significant changes in mitochondrial membrane lipidome. These changes include (1) a decrease in the relative levels of phosphatidylethanolamine (PE), cardiolipin (CL) and monolysocardiolipin (MLCL) within mitochondrial membranes; and (2) an increase in the relative levels of phosphatidic acid (PA), phosphatidylserine (PS), phosphatidylcholine (PC) and phosphatidylglycerol (PG) within mitochondrial membranes (Fig. 1). In turn, the LCA-driven changes in the composition of mitochondrial membrane phospholipids cause: (1) a substantial enlargement of mitochondria; (2) a significant decrease in mitochondrial number; (3) a reduction in the fraction of mitochondria with cristae extending from the IMM; and (4) a build-up within the mitochondrial matrix of abundant cristae disconnected from the IMM (Fig. 1). These elicited by LCA major alterations in mitochondrial abundance and morphology in turn cause specific changes in the age-related chronology of such longevity-defining redox processes confined to mitochondria as respiration, the maintenance of mitochondrial membrane potential, the preservation of cellular homeostasis of mitochondrially produced ROS, and the coupling of electron transport to ATP synthesis (Fig. 1). The elevated efficiencies of mitochondrial respiration, membrane potential maintenance and ATP synthesis observed in chronologically “old”, quiescent yeast cultured with LCA (as compared to those in age-matched cells cultured without LCA) are known to delay aging of yeast cells by increasing their long-term viability [18–23]. Furthermore, the raised sub-lethal concentration of mitochondria-generated intracellular ROS detected in this yeast (Fig. 1) has been demonstrated to delay cellular aging because it stimulates a signaling network promoting the long-term stress resistance of yeast cells [5–7,18–21]. Importantly, the longevity-extending pattern of mitochondrial redox processes characteristic of chronologically “old”, quiescent yeast cultured with LCA (Fig. 1) is likely due to the lowered below a toxic threshold intracellular concentration of ROS seen in chronologically “young”, non-quiescent yeast cultured in the presence of this bile acid [18,19]. Indeed, a reduced ROS level in chronologically “young” yeast cells exposed to certain anti-aging interventions has been shown to lessen the extent of oxidative damage to mitochondrial macromolecules in these cells, thereby allowing them to maintain the functionality of macromolecules involved in mitochondrial respiration, membrane potential maintenance and ATP synthesis when these cells become chronologically “old” [18,19,21,23]. Ultimately, the elicited by LCA changes in the age-related chronology of these mitochondrial redox processes extend longevity of chronologically aging yeast.

## Figures and Tables

**Fig. 1:**
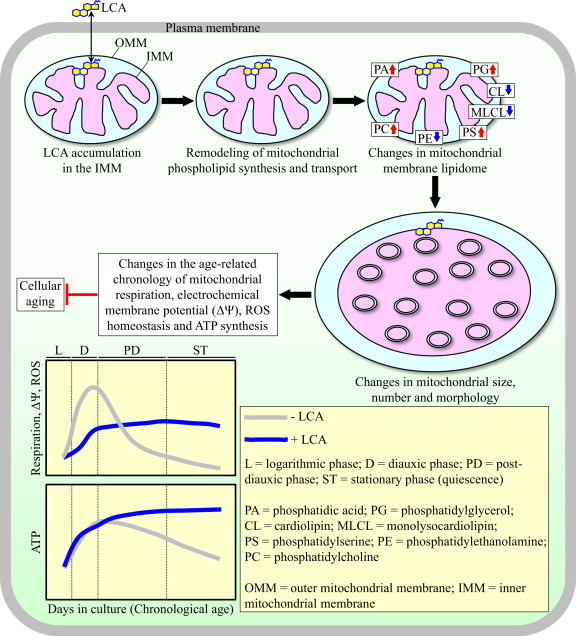
A mechanism of delaying aging in yeast by a mitochondrially targeted compound which impacts mitochondrial redox biology. Lithocholic bile acid (LCA) enters yeast cells, accumulates mainly in the inner mitochondrial membrane, and elicits a remodeling of phospholipid synthesis and movement within both mitochondrial membranes. Such remodeling of mitochondrial phospholipid dynamics causes changes in mitochondrial membrane lipidome. These changes in the composition of membrane phospholipids alter mitochondrial abundance and morphology, thereby triggering changes in the age-related chronology of several longevity-defining redox processes confined to mitochondria. For additional details, see text.
